# Decision Support Systems in Neurosurgery: Current Applications and Future Directions

**DOI:** 10.3390/s25247415

**Published:** 2025-12-05

**Authors:** Mateusz Koryciński, Konrad A. Ciecierski, Ewa Niewiadomska-Szynkiewicz

**Affiliations:** Institute of Control and Computation Engineering, Warsaw University of Technology, Nowowiejska 15/19, 00-665 Warsaw, Poland; mateusz.korycinski@pw.edu.pl (M.K.); konrad.ciecierski@pw.edu.pl (K.A.C.)

**Keywords:** neurosurgery, decision support systems, artificial intelligence, MRI, data analysis

## Abstract

Artificial intelligence (AI) is one of the fastest-developing research fields. Its methods are applied with great success to various problems across many industries. Healthcare is one of them, with AI applied to organizational problems, textual data analysis, and treatment decision support systems used for diagnosis and treatment. This paper reviews current AI methods in neurosurgery settings, discussing the potential use of decision support systems. As neurosurgery is highly technical and requires millimeter-precise guidance, AI systems can provide significant benefits in helping neurosurgeons navigate the surgical field. For example, AI-assisted neuronavigation during deep brain stimulation (DBS) surgeries shortens the length of the procedure and significantly reduces the risk of electrode misplacement, and the need for future costly re-operation. We do not limit our review to existing methods, as we also discuss possible future directions for such systems. When developing such methods, special emphasis must be put on precision, usability, security, and explainability.

## 1. Introduction

As life expectancy continues to rise globally, healthcare systems are increasingly confronted with a growing burden of chronic conditions, including neurological disorders. These disorders now represent the leading cause of disability and the second leading cause of death worldwide [1]. Many neurological conditions can be managed or alleviated through surgical intervention, making neurosurgery a critical component of modern healthcare.

Neurosurgical procedures vary widely depending on the underlying pathology and can be broadly categorized into neuro-oncology, vascular, functional, spinal, traumatic brain injury (TBI), emergency, and skull base surgery. Neuro-oncology focuses on tumors affecting the central nervous system, including both primary tumors (e.g., gliomas, meningiomas) [2] and metastatic lesions originating from cancers such as lung or breast [3]. The goals of neuro-oncological surgery range from diagnostic biopsy and tumor resection to palliative interventions aimed at relieving symptoms such as elevated intracranial pressure or neurological deficits [4].

Despite advances in surgical techniques, neuro-oncological procedures remain clinically and technically challenging. Accurately delineating tumor margins is particularly difficult, as many tumors infiltrate eloquent brain regions and critical neural pathways. While gross total resection may offer the best chance of tumor control, it often risks impairing essential cognitive or motor functions. As a result, subtotal resection is usually performed, which may leave residual tumor cells and increase the risk of recurrence [5]. To optimize surgical outcomes, technologies such as intraoperative magnetic resonance imaging (MRI), neuronavigation, and awake brain mapping are employed [6,7]. However, these tools are not universally available, limiting access to precision-guided neurosurgery in many settings.

Vascular neurosurgery addresses abnormalities of the cerebral vasculature, including aneurysms and arteriovenous malformations (AVMs) [8]. These conditions pose serious risks to the central nervous system, potentially leading to hemorrhagic or ischemic stroke, seizures, or even sudden death. Timely and effective surgical interventions—such as microsurgical clipping, AVM resection, or cerebral bypass—are essential to prevent rupture, restore cerebral perfusion, reduce seizure burden, and preserve neurological function [9]. However, these procedures are technically demanding and often performed under high-risk conditions, where even minor errors can result in devastating outcomes. The decision-making process is multifaceted, requiring integration of clinical presentation, imaging data (e.g., computer tomography (CT) angiography, digital subtraction angiography, perfusion MRI), and risk stratification models [10]. This allows for effective consideration of factors such as lesion size, location, vascular architecture, and patient comorbidities.

Functional neurosurgery is a field aimed at alleviating and improving the quality of life for patients with neurological conditions such as movement disorders (e.g., Parkinson’s disease, essential tremor), epilepsy, and chronic pain. These interventions work by modulating the electrical or chemical activity of specific brain or spinal cord regions. Common techniques include deep-brain stimulation (DBS), spinal cord stimulation (SCS), and stereotactic lesioning.

In DBS, electrodes connected to a pulse generator are implanted into targeted brain areas to deliver controlled electrical impulses. This allows for modulating and effectively treating abnormal neural activity causing disorders that are usually resistant to traditional treatment, such as Parkinson’s disease, dystonia, tremor, depression, and OCD [11]. SCS is a similar technique that targets the spinal cord to manage chronic neuropathic pain by disrupting pain signal transmission. Stereotactic lesioning uses a different approach to the modulatory effect. By adapting techniques such as radiofrequency ablation [12] or focused ultrasound [13], small, precise lesions are introduced in the neural tissue to interrupt dysfunctional circuits. This technique is often employed when stimulation is ineffective or impractical.

All these procedures require careful and time-consuming planning, often based on imaging techniques (e.g., MRI, CT) and intraoperative neurophysiological monitoring. In recent years, additional information from connectomics and functional magnetic resonance imaging (fMRI) has also been included to refine planning and improve outcomes.

Similar to other types of neurosurgery, high-resolution MRI and CT are essential for preoperative planning. Intraoperative support systems, including neuronavigation, endoscopy, and neurophysiological monitoring, facilitate more accurate and safe procedures.

Each neurosurgical subspeciality presents distinct challenges across the continuum of care—from diagnosis and surgical planning to intraoperative decision-making and postoperative management. A common task among these challenges is the need to synthesize vast amounts of heterogeneous data, including clinical records, imaging studies, physiological monitoring and intraoperative findings. Making informed decisions under such conditions requires not only clinical expertise but also efficient tools for data integration and interpretation.

To address this complexity, computer-based decision support systems (DSSs) have been developed to assist and support clinicians and medical professionals in making timely, evidence-based, and data-driven decisions throughout the treatment process [14]. In general, DSSs integrate data analysis, modeling, and visualization techniques to facilitate complex decision-making processes. They have been widely adopted across various domains, including finance, agriculture, environmental management, logistics, manufacturing, and defense. In the financial sector, DSSs play a crucial role in strategic planning, risk assessment, or investment analysis, enabling institutions to respond swiftly to market dynamics [15,16]. In modern, sustainable agriculture, DSSs support crop planning, irrigation scheduling, and pest control [17,18]. Similarly, in environmental management, DSSs are employed to monitor pollution, guide mitigation strategies, and allocate natural resources more efficiently [19,20,21].

The complexity of such tools can vary significantly, depending on their intended application and the nature of the data they are designed to process. Each system is typically designed to extract information from a specific type of data, which may include numerical values, structured tabular datasets, or unstructured formats such as medical images. DSSs incorporate a range of analytical techniques to effectively handle such diverse data modalities, from classical mathematical models to advanced machine learning (ML) algorithms.

Traditional approaches often rely on statistical methods and rule-based logic [22], while modern systems increasingly leverage ML models such as support vector machines (SVMs), decision trees, and ensemble methods. Among these, neural networks—particularly deep learning architectures—have garnered substantial attention due to their ability to learn complex patterns from large and heterogeneous datasets. This shift toward data-driven, adaptive models has significantly enhanced the predictive power and versatility of DSSs, especially where decision-making relies on integrating multimodal information.

The versatility and adaptability of DSSs across such diverse domains underscore their potential value in healthcare, and more specifically, in neurosurgery, where decision-making is often time-sensitive, data-intensive, and critically consequential. Given the complexity and high stakes involved in neurosurgical procedures, DSSs can play a pivotal role in enhancing clinical accuracy, reducing variability, and supporting evidence-based practices.

Due to the broad scope of neurosurgery, a wide range of DSSs have been developed, each tailored to specific surgical contexts and clinical needs. We have organized this section according to the major surgical types outlined in the introduction to provide a clear and structured overview. This approach allows for a more focused analysis of how these systems are integrated into different neurosurgical workflows and decision-making processes.

The scope of the present review is broad. We intended this paper to highlight domains of neurosurgery where computer-based systems can effectively support medical professionals in decision-making. Therefore, we review a selected applications, with a focus on machine learning methods that can serve as a basis for a decision support system in neurosurgery. The following sections provide an overview of machine learning and deep learning solutions in DSSs applied for specific tasks within various neurosurgical domains. We highlight their current capabilities, limitations, and potential for future integration into clinical workflows.

In selecting the papers included in this review, we focused on highlighting significant papers for each of the described branches of neurosurgery. These were not always the newest papers, but we tried to select ones that show advances in problems that still exist and require addressing. One of the attributes considered during the paper selection process was the number of citations, which may indicate the significance of the solution.

The structure of this paper is as follows. Section 2 focuses on DSSs’ applications in neuro-oncology, highlighting their use in diagnostic workflows as well as in preoperative and intraoperative settings. Section 3 examines decision support methods in vascular neurosurgery, with particular attention to intracranial aneurysms, which represent a key area of application. Section 4 explores DSSs in functional neurosurgery, discussing current approaches and emerging trends. In Section 5, we emphasize the need for continued methodological advancement, particularly through the development of more sophisticated architectures such as multimodal classifiers. This section also outlines how a comprehensive DSS could provide visual guidance to neurosurgeons, integrating diverse data sources into a unified interface. Finally, Section 6 presents a summary and discussion, synthesizing key insights and outlining future directions for research and clinical implementation.

## 2. Decision Support in Neuro-Oncology

Neurology and neurosurgery are among the newest specialties in medicine. Their development required prior discoveries in scientific research and technological advancements. Among technologies that contribute greatly to these specialties are machine learning (ML) and, recently, the AI revolution. Many neurosurgical procedures, such as stereotactic surgery, deep brain stimulation, or cortical mapping in search of Seizure Onset Zones, would not be possible without advanced technologies. AI has many applications in neuro-oncology, starting with diagnostic medicine, followed by various aspects of neurosurgery, post-operative intensive care, pathophysiology, cancer genetic profiling, and planning of post-operative treatment [23].

### 2.1. Diagnostic Application

At the start of the treatment of oncological disease, the first step must be detailed diagnosis followed by a decision about the treatment. The diagnosis is mainly based on the results of medical imaging, such as CT and MRI. Its role, among others, is to assess the extent of the tumor, determine the feasibility of various neurosurgical interventions, consider the risks associated with different approaches, and ultimately plan the neurosurgical procedure itself. For example, certain decision support systems can predict whether the tumor exhibits characteristics typical of specific mutations, which in turn can be helpful when planning surgery.

#### 2.1.1. Tumor Detection and Classification

**Figure 1:**
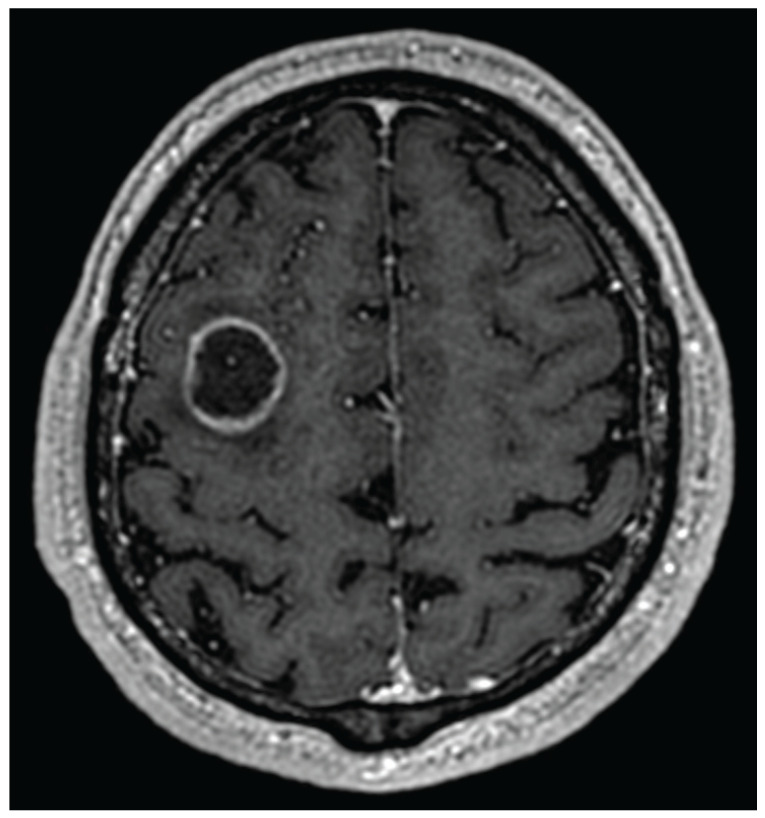
Glioblastoma with ring enhancement on MRI T1 with contrast.

In case of suspicion of a brain tumor, the first diagnostic methods are computer tomography and magnetic resonance imaging. These two methods provide good information about the abnormalities in nervous system anatomy. Reading of such images is, however, not an easy task, and for that reason, there is a separate medical specialization—radiology. Various brain tumors appear differently depending on the MRI modality used or the contrast medium employed. The most common and also most aggressive primary brain tumor—glioblastoma—may look very different in CT/MRI images due to its high mutation potential. The typical glioblastoma contrast ring enhancement (see Figure 1) might also be present with other brain tumors, while one might also observe glioblastomas without such an enhancement [24]. While the final diagnosis is always based upon histopathology and cancer genetic profiling, medical imaging provides strong clues as to what type of disease is present, what kind of treatment should be applied, and whether, for example, an additional brain biopsy is required for further diagnosis.

In recent years, artificial intelligence techniques have dominated the detection and classification of brain tumors. The proposed solutions vary in terms of the complexity of the models used and the number and diversity of the datasets on which they were trained.

Jia et al. [25] used image preprocessing, segmentation, and feature extraction to identify and determine the boundary of the tumor. The authors separated the whole cerebral venous system by MRI imaging using a novel algorithm based on structural, morphological, and relaxometry details. Finally, the SVM binary classifier was created based on the extracted features [26]. They achieved an accuracy of 98.51% in detecting abnormal and normal tissue in MRI images. Similar to [25], Anantharajan et al. [27] proposed a binary classifier that states whether a given MRI slice contains a tumor or not. They used the gray-level co-occurrence matrix (GLCM) to extract features (e.g., energy, mean, entropy, contrast). Finally, they constructed an ensemble deep neural support vector machine (EDN-SVM) to identify abnormal tissues. The model was evaluated on a dataset taken from the Kaggle website. It included 255 T1-mode MRI images, comprising 98 slices from healthy brain tissue and 155 slices from tumorous brain tissue. The achieved detection accuracy was 97.93%, sensitivity of 92%, and specificity of 98%. In [25,27], the only information returned was whether the slice contained a tumor or not. No tumor type or its location is provided by the network, making serious clinical applications difficult.

In [28], the authors use a combination of stationary wavelet transform (SWT), Random Forest (RF) classifier, and growing convolutional neural network (GCNN) for segmentation and detecting tumor boundaries. The model was trained on BRAINX (https://brainxai.org/data/) medical images. The performance of the proposed solution was compared with common machine learning techniques. The authors claim that their technique outperforms K-NN, SVM, self-organizing map (SOM), CNN, and genetic algorithm (GA). The achieved sensitivity and precision were 98.23% and 98.81%, respectively.

The authors of the three following papers [29,30,31] applied artificial neural networks for the classification of the three most common types of brain tumors, i.e., glioma, meningioma, and pituitary tumor. Abiwinanda et al. [29] constructed and evaluated a CNN-based classifier. An entire slice of an MRI scan (512 × 512) is used as input, and the model returns a classification result. The architectures of the described neural networks are simple, and no bounding box of the tumor is being returned—only one of three possible classes. The CNN architecture was trained and validated on images from the brain tumor dataset (Figshare [32]), which comprises 3064 T1-weighted contrast-enhanced MRI images: meningioma (708), glioma (1426), and pituitary (930). The images were acquired from 233 patients at Nanfang Hospital and General Hospital, Tianjin Medical University, China, between 2005 and 2010. They achieved a training accuracy of 98.51%, and validation accuracy of 84.19%. However, it is worth noting that classification is straightforward; for example, all types of gliomas are grouped into a single standard class, which is of limited clinical utility.

Badža et al. [30], similarly to [29], use CNN for the classification of the MRI slices into the same types of brain tumors. The network input data differs slightly. The slices are in lower resolution (256 × 256), but each tumor is shown on three slices (axial, coronal, and sagittal). The authors used the same base dataset [32] for training and testing the network. They increased the collection of available images threefold by applying common data augmentation techniques (rotation by an angle of 90 and flipping vertically). Ultimately, they obtained 9192 images. The best 10-fold cross-validation accuracy for the augmented dataset and record-wise approach was 96.56%. For the subject-wise approach, the accuracy was 88.48%. It is worth noting that, due to the simple CNN architecture, the model can be deployed on mobile platforms.

In [31], the authors propose more advanced neural network architectures to classify the brain tumor type. They apply transfer learning techniques using natural images of the ImageNet dataset [33], and identify the brain tumor type using the Figshare dataset. Three CNN architectures were compared: AlexNet, GoogLeNet, and VGGNet. Similar to [30], they applied rotations (angles of 90, 180, 270) and flipping (vertical and horizontal direction) to increase the training dataset. Using the fine-tuned VGGNet (VGG16) [34], they obtained a test accuracy of 98.69%. Still, as all glioma types are grouped together in a single class, this solution, like the one discussed above, has limited application in clinical medicine.

In [35], the authors used a 3D-ResNet101 [36,37] deep learning model on volumetric MRI data for tumor grading classification. The network architecture was trained and validated on images acquired from 919 patients with glioma from the Second Hospital of Lanzhou University and the TCIA database [38]. The accuracy achieved by this binary classifier was 83%, F1 score was 83%, and AUC was 89%. The authors claim that 3D-CNN outperforms 2D-CNN. While the accuracy is good, unfortunately, one finds only discrimination between high-grade and low-grade brain tumors, which itself is a rudimentary task for a radiologist.

Table 1 summarizes the discussed AI techniques that support tumor detection and classification.

A comprehensive review on brain tumor segmentation and classification of MRI images is provided in [39,40,41]. Moreover, many papers focus on the advancement of computer vision in tumor detection, leaning heavily toward the computer science aspect rather than clinical applicability. The solutions they provide certainly advance research in computer science, but their clinical applicability is unfortunately limited.

In this context, it is worth mentioning the prestigious MICCAI conference, organized by the Medical Image Computing and Computer-Assisted Intervention Society. This conference focuses on solutions that advance scientific knowledge in both computer science and the medical sciences. One of the important MICCAI initiatives is the Brain Tumor Segmentation (BraTS) Challenge [42]. There is also a branch of BraTS that, since 2023, has been focused on pediatric brain tumors [43]. During the last few years, the BraTS initiative has collected an extensive database of brain tumors that can be used for the development, training, and testing of various methods of detection and segmentation of brain tumors. The BraTS initiative also annually provides benchmarks for the best tumor analysis methods.

This initiative addresses the obvious need for tools to effectively discriminate between various grades of gliomas, as this significantly influences potential treatment approaches and the patient’s outlook for survival. This is especially important in children, as the tumors of the central nervous system are the most common cause of cancer-related death among them.

#### 2.1.2. Radiomics

Radiomics is a field of clinical medicine focusing on in-depth analysis of data acquired from medical imaging techniques, e.g., MRI. Radiomics involves such tasks as image preprocessing, segmentation, feature extraction, data analysis, knowledge extraction, and finally clinical application of such knowledge. It focuses on the extraction of meaningful features that are not directly or easily visible in medical imaging data.

In [44], the authors use radiomics features to predict if the tumor is of WHO class 2, 3, or 4. Radiomics features are extracted from a single T2-weighted MRI slice using the texture analysis research software TexRAD Version 3.10. The number of features is then decreased using recursive feature selection. Finally, a binary classifier is applied to discriminate the examined glioma into WHO class 2, 3, or 4. The best results were achieved using the Random Forest classifier with an AUC score of 0.81 and an F1 score of 88%. While these results are clinically significant, the solution nonetheless requires the manual use of specialized, licensed software for essential feature extraction.

In [23], the authors describe the application of AI in determining candidates for more radical resection of gliomas. Discrimination was performing using machine learning on classical and deep radiomic attributes. The first classifier was an SVM trained on radiomic data to discriminate between high-risk patients (those with a life expectancy of less than 6 months) and low-risk patients. The second SVM classifier identified patients with a lower risk and a life expectancy of more than 18 months. The results of the classification showed a group of patients for whom total or near-total tumor resection was associated with a more prolonged overall survival. Still, one must be aware that a patient for whom a total or near-total resection was not undertaken has a tumor of a greater extent involving brain regions that, for neurophysiological reasons, could not have been resected. This, in itself, also influences the outcome of the analysis.

#### 2.1.3. Radiogenomics

Radiogenomics is a branch of clinical research that builds upon the results obtained by applying radiomics. Its goal is to predict the genetic characteristics of a tissue visualized by the medical imaging techniques based on the information provided by the radiomics.

In [45], the authors use machine learning to predict PTEN [46] gene mutations and deletions. Mutation and especially deletion of this gene are pivotal for the occurrence of the most severe brain tumor, Glioblastoma IDH-wildtype. The PTEN gene acts as a tumor suppressor gene in a cell, and its reduced activity or lack thereof is strongly oncogenic.

#### 2.1.4. Summary

While there are many decision support systems for tumor detection in the MRI data, they have various limitations. Some limitations are clearly due to the lack of a well-annotated dataset. This can be observed in many papers that discriminate only between three types of brain tumor, i.e., glioma, meningioma, and pituitary tumor. While such approaches have merit from the computer science point of view, their practical clinical applicability is limited as it is very easy to distinguish between these tumors without any help from a decision support system [47]. Other approaches rely on licensed paid software or focus solely on gliomas. It is evident that there is a need for further development of decision support systems in neurological oncology diagnostics, particularly for comprehensive tools that can detect, localize, and classify brain tumor types and histological subtypes.

### 2.2. Decision Support in Preoperative Planning

After the initial diagnosis using the methods described in the previous section, the neurosurgeon can determine the type, size, and location of the tumor. This information initiates the preoperative planning process, which must consider not only the technical feasibility of the surgical approach but also the preservation of critical neurological functions. The principle of primum non nocere—first, do not harm—remains central to neurosurgical decision-making. In some cases, the surgical approach itself or the extent of resection required may pose a high risk of neurological damage, rendering certain tumors effectively inoperable.

To minimize these risks, additional functional and structural information is gathered using advanced MRI modalities, particularly functional MRI (fMRI) [48] and diffusion-weighted imaging (DWI) [49]. fMRI allows visualization of functional cortical areas adjacent to the tumor, responsible for language, motor control, speech production and perception, or sensory processing. Damage to these eloquent areas can result in significant impairments affecting the patient’s quality of life. Accurate mapping of these regions allows the surgical team to balance the extent of tumor resection with the preservation of neurological functions. In many cases, a subtotal resection that spares eloquent cortex is preferred over a more aggressive approach that risks permanent deficits.

Interestingly, due to brain plasticity, certain functional areas may shift slightly over time [50]. In the case of slow-growing tumors, this allows neurosurgeons to stage treatment over time and perform more extensive resections in subsequent procedures without compromising functions. Thus, preoperative planning is not a static process but one that must adapt to individual patient anatomy and functional organization.

Task-based fMRI is a powerful diagnostic tool that enables the identification of specific brain regions activated during controlled tasks performed by patients during an MRI scan. In [51], Kuan et al. applied machine learning and deep learning to voxel-wise classification of an fMRI signal from brain regions subserving language. Their methods were trained on the data from six tasks performed by fourteen healthy individuals and tested on six tasks performed by six healthy individuals and six epilepsy patients. The results they achieved are outstanding. The best area under the ROC curve (ROC AUC) of 0.97 ± 0.3 with a Dice coefficient of 0.6 ± 0.34 was achieved with a Rotation Forest algorithm [52]. The second best was an interval-based method—a Supervised Time Series Forest [53]—which achieved a ROC AUC of 0.96 ± 0.03 and Dice coefficient of 0.61 ± 0.33. The deep learning approach, namely the Inception Time method [54], was not as good, with an ROC AUC of 0.94 ± 0.04 and Dice coefficient of 0.52 ± 0.34. The method was developed for a block-designed fMRI, and according to the authors, it shall be applicable to experiments with a similar design. Further research is required, however, to investigate how this method generalizes to other block designs. To the best of our knowledge, this is the first and only method that utilizes machine learning and deep learning to map functional cortical areas from task-based fMRI data. Evidently, although time-consuming, classical approaches to analyzing such signals are mostly adopted by radiologists.

Functional brain mapping can also be achieved by resting-state fMRI (rs-fMRI). In contrast to a task-based fMRI, it does not require a patient to perform any tasks, thereby eliminating the need to design complex experimental paradigms [55]. Hence, it is applicable to children who are too young to perform certain tasks properly (especially infants), cognitively impaired patients of any age, or sedated patients. Machine learning methods for analyzing rs-fMRI signals are often used to detect and classify neurodevelopmental disorders, such as ADHD [56] or autism [57]. However, even though performed in a task-negative state, it also allows for mapping functional connectivity of the cortex as shown by Biswal et al. nearly thirty years ago [58]. Furthermore, in recent years, we have observed the development of machine learning and deep learning methods crafted for this task to support the data analysis process. In [59], Luckett et al. trained a 3D convolution model consisting of a total of 60 layers to classify each voxel from an rs-fMRI experiment to either motor, language, or other resting-state network. Their model achieved 96% accuracy on a test set comprising scans from both healthy adults and five subjects with glioblastoma. The model was trained on either 50 or 200 rs-fMRI time points (∼2.5 or ∼10 min acquisition time), showing 97.9% similarity between these variants. Particularly interesting are the results from the glioblastoma subjects, where the model was able to correctly classify voxels and accurately follow the structural alterations caused by the tumor, even when a shorter acquisition time was used. Such an approach can provide valuable insights into preoperative planning, thereby decreasing patient discomfort caused by a longer acquisition time.

In addition to functional MRI (fMRI), tractography provides a valuable source of information for neurosurgical planning, offering critical insights into the brain’s structural connectivity [60]. Tractography is an advanced imaging technique, derived from diffusion-weighted imaging (DWI), which enables the visualization and reconstruction of white matter tracts—neural pathways leading to functional cortex areas. Damage to the neural fibers can be detrimental to the functions provided by the cortex area if it is disconnected from the neural circuits. Tractography is a hard task, given the complex characteristics of the diffusion signal. Although various methods for analyzing DW-MRI signals exist, they often generate spurious tracts, resulting in a high level of false-positive results. Often, AI methods are designed in a black-box approach, where the neural network computes the final classification result based on the analysis of the input data.

Researchers in tractography use machine learning and deep learning heavily to interpret various DWI sequences. The first attempts utilized classical machine learning models like Random Forest with classifier voting [61], AdaBoost [62], or support vector machines [63]. With the rapid advancement of deep learning, neural networks have garnered increasing attention for analyzing DWI signals. Initial models were rather simple and utilized fully connected layers [64,65,66], or convolutional layers [67,68,69,70]. Recent works emphasize a hybrid approach, combining various architectures into a single network, or fusing neural networks with other methods.

In [71] Kumar et al. introduce BrainTract, a hybrid DISAU-Net architecture combining an Inception-ResNet-V2 module with a Dense-Inception block into a Spatial Attention U-Net. Inception-ResNet-V2 module replaces standard convolutional layers, allowing for expansion of the model width, while Dense-Inception block extracts features and deepens the network without the need to introduce more parameters. The model achieved an accuracy of 97.10% and a Dice score of 96.88%.

In [72] we introduced a hybrid tractography method HyTract, where the neural network comprising four fully connected layers determines a graph of probable connections that are further interpreted by the path search algorithm A* to compute neural tracts. A small number of parameters makes the inference time shorter and allows training on a regular GPU with limited memory. Despite its simplicity, this method achieves a ROC AUC of 0.97 for a validation dataset and correctly identifies tracts originating in the visual cortex, outperforming other methods in the test dataset. Table 2 summarizes the discussed AI techniques supporting preoperative planning.

### 2.3. Decision Support During Neurosurgical Procedures

During neurosurgical procedures, decision support systems are used, among others, for neuronavigation and anesthesiological monitoring of the patient.

#### 2.3.1. Intraoperative Neuronavigation

During a neurosurgical procedure, the brain tissue swells and shifts. As the goal of surgery is the maximal but safe resection of the tumor, these changes make achieving this goal even more difficult. Without taking this into account, there is an increased risk of error during the surgery, and in the brain, what is cut unfortunately stays cut. The preoperative imaging may no longer reflect the actual situation, and to update information about the surgical field, additional medical imaging is performed during the surgery itself. While intraoperative MRI gives the best results, it is not always available and considerably prolongs the neurosurgical procedure, increasing the physiological stress on the patient’s body. Because of this, other medical imaging techniques are often utilized during surgery, and their results are used to update the anatomical information from the preoperative MRI. For this reason, there are machine learning-based solutions that help fuse medical imaging from various modalities intraoperatively.

In [77], the authors provide a case study based on four patients with brain tumors. The preoperative MRI is augmented/fused with data obtained from a computer tomography and intraoperative ultrasound during surgery. The fusion is performed using a commercial neurosurgical software package, Brainlab Elements 4.0.

The AI solutions embedded in Brainlab enable the rapid, automatic fusion of medical image data, allowing neurosurgeons to perform intraoperative ultrasound repeatedly during resection without significantly prolonging the surgery. In conclusion, the authors emphasize that while AI aids during surgery, the surgeon must not blindly trust the virtual visualizations provided by the AI.

In [78], the authors compare the standard neuronavigation with neuronavigation augmented by intraoperative ultrasound and MRI. Comparison is based on surgeries for patients with diffuse gliomas. In the study, 80 patients who underwent standard neuronavigation were compared to 80 patients who underwent neuronavigation augmented by intraoperative ultrasound and MRI. The authors used AI embedded in the Sinovation Medical Technology software package. The use of AI significantly increased the time of the surgery without a corresponding improvement in long-term postoperative neurological deficits. However, it noticeably increased the survival time in patients with glioblastoma.

In [79], the authors divided 108 glioma patients into two groups. The control group underwent classical tumor removal, while the experimental group had their tumors resected with support from intraoperative MRI. The data from intraoperative MRI was analyzed during the surgery using a segmentation dictionary learning algorithm.

#### 2.3.2. Stereotactic Biopsy Guidance

In cases where medical imaging techniques detect pathological tissue in the brain, a brain biopsy is often performed to obtain histopathological information. The problem (similar to one present in deep brain stimulation, described later in this section) is that to reach the point from which the biopsy is to be taken, one must first pass through the brain parenchyma. Brain tissue makes us who we are, so it is extremely precious; therefore, the biopsy trajectory cannot be random and must be chosen carefully to avoid certain structures, such as larger arteries or veins.

In [80], the authors use MRI T1 sequence image data together with the Magnetic Resonance Angiography T2 sequence to suggest the biopsy entry points on the patient’s skull that are best suited and safest to reach the selected target. The algorithm first distinguishes and segments the tumor, ventricles, arteries, and veins. For this purpose, the authors use the 3D Residual U-Net architecture. Finally, all possible entry points are presented to the neurosurgeon for their final decision.

#### 2.3.3. Intraoperative Brain Mapping

When brain surgery is near the eloquent areas of the cortex, i.e., areas specialized in certain functions, such as speech or movement, these areas are mapped during the surgery to preserve their functionality to the maximum possible extent. One of the methods used to detect such eloquent areas is the detection of the cortico-cortical evoked potentials. In this method, one area of the cortex is electrically stimulated, while another is observed for the occurrence of the evoked potentials. If they are detected, it is a strong indication that these two cortical areas are neurophysiologically connected and perform similar functions. Additional information is also provided by the amplitude and the latency of the recorded evoked potential.

In [81], the authors present a pilot study to predict post-operative speech deterioration using machine learning analysis of the cortico-cortical evoked potentials (CCEp). The stimulating electrode strip was placed on Broca’s area, and the strip recording evoked potentials was placed on different parts of the upper temporal gyrus where surgery took place. The authors trained various models to predict speech deterioration using the recorded data and post-surgical evaluation. While the obtained results are not excellent (the best F1 score is 64% with an AUC of 0.68), this paper demonstrates that there is potential for future machine learning applications in the analysis of CCEp.

#### 2.3.4. Summary

With advances in the field of neurosurgery, an increasing number of decision support systems are being utilized during surgical procedures in the operating theater. Neuronavigation, stereotaxy, neurophysiology, anesthesiology, and many other medical fields benefit from the application of machine learning and AI. In some areas, numerous applications are already in use, while in others, initial attempts are being made, and although the results are interesting, they are not yet satisfactory. Already, it is hard to imagine many neurosurgical procedures without the aid of computer systems, and one can be certain that, over time, cooperation between medical sciences and computer sciences will only increase.

### 2.4. Decision Support in Post-Operative/Traumatic Intensive Care

Neurosurgery is a very invasive procedure on the most fragile organ of the human body, on “The Fragile House of the Soul” (Jürgen Thorwald). After such procedures, people frequently need intensive post-operative care and monitoring. Surgical intervention causes the brain to swell, and that in turn increases the intracranial pressure (ICP), which, when not properly maintained, can be very harmful or fatal. It is essential not only to monitor the ICP but also to be able to predict its increase, thereby preventing potentially life-threatening situations.

In [82], the authors use an LSTM network to predict the occurrence of increased ICP in the time horizon reaching up to 24h. Features used for prediction included 26 vital signs, 36 laboratory tests, seven medications, and 16 blood gas analysis results. The model was trained to predict the occurrence of short or long increased ICP phases. The short phase consisted of up to two critical hours, and the long phase consisted of more than two, where a critical hour was defined as an hour during which the ICP was measured at 22 mmHg or higher at least once. When the prediction horizon was one hour, the AUC for the test data from Medical Information Mart for Intensive Care [83] was 0.965. For the 24-h horizon, the AUC was 0.836.

In [84], the authors use machine learning to predict the approaching ICP crisis using readouts from the intraparenchymal fiber-optic device in patients with severe traumatic brain injury (GCS<8) [85]. An ICP crisis is defined in the paper as an ICP over 22 mm Hg for at least 75 percent of the data collected within a 5-min interval. The task for classification was to predict, using ICP measurements from a one- or two-hour-long window, that an ICP crisis would occur within 10 or 20 min following it. The best obtained AUC value was between 0.86 and 0.88, depending on the chosen dataset, when using the Random Forest classifier.

Post-operative ICP monitoring or monitoring in patients with severe traumatic brain injury can successfully be augmented with the application of machine learning. The ability to predict the approaching ICP event is crucial, as it gives doctors time to administer proper treatment that will alleviate or prevent such an event. This is literally life-saving as an ICP crisis lasting five minutes or more radically reduces the chances of a positive outcome [84].

### 2.5. Decision Support in Histopathology and Cancer Genetic Profiling

After the surgery, or sometimes even during it, when the tumor mass is removed, it is pathophysiologically inspected and classified. In recent years, pathophysiology has also been routinely accompanied by clinical genetic testing for various oncogenic mutations. For example, a tumor whose pathophysiological picture matches glioblastoma is finally graded as glioblastoma only if it lacks IDH mutation (called glioblastoma IDH-wildtype). If the mutation is present, it is classified as an astrocytoma IDH-mutant WHO grade 4. This distinction is not only theoretical, as these two tumors, while both are ultimately fatal in most cases, have very different mean survival rates. Patients with astrocytoma IDH-mutant WHO grade 4 may live up to three years or more, while those with glioblastoma IDH-wildtype rarely survive 15 months.

#### 2.5.1. Applications in Histopathology

One of the earliest applications of deep learning was image recognition. It is not surprising that computer neural networks find their use in histopathology, where the goal is the detection and classification of histological abnormalities.

In [86], the authors use transfer learning to classify images of histological H&E-stained preparations of brain tissue into two or three classes. In the case of two-class classification, these classes are normal brain and high-grade glioma. In the case of three-class classification, the classes are normal brain, low-grade glioma, and high-grade glioma. As a classifier, the authors use a deep neural network based on the Inception V3 [87] network by means of transfer learning. For the two-class problem, the network achieved perfect classification of the test data. In the case of three-class classification, both recall and precision scores were above 94%.

In [88], the authors focus on the classification of histological samples intraoperatively, i.e., during the surgery itself. H&E staining provides good visualization for histopathological classification, but it takes days to properly stain the sample, making this approach unfeasible for application during surgery. Intraoperative knowledge can be of great importance to the neurosurgeon as it may influence the extent of the surgery. The authors of this paper develop an image processing technique that produces virtual H&E-stained views of unstained, intraoperatively acquired samples. These virtual images are then classified in a similar way to the stained samples. Classification into three classes, i.e., normal brain tissue, low-grade glioma, and high-grade glioma, achieved an accuracy of 0.9.

Another noteworthy AI approach for analyzing images of histological samples is a dual-channel network for pathology image classification, described in [89]. This approach facilitates the transfer learning-based strategy called Few-Shot Learning (FSL). Here, the deep learning-based classifier is trained on a certain base dataset, and the resulting classifier is fine-tuned for the target classification task using only a limited number of training samples. Such an approach can be beneficial for classifying various histological types of brain tumors.

In many cases, the assessment of histological samples must be performed during the surgery itself, as the diagnosis may influence the extent of the remaining surgery. If the glioma is of a lower grade, the surgeon might aim for a total or near-total resection, while in the case of GBM, the surgery might be continued in a more palliative direction. For this reason, approaches that allow histopathologists to make a rapid diagnosis are beneficial. One such approach is described in [90]. Here, the model was trained using an eye-tracking device worn by a histopathologist during the assessment of whole-slide images. The model trained in this way assists histopathologists during the diagnosis of new slides. In this way, the time needed for histological assessment is radically reduced.

#### 2.5.2. Applications in Genetic Testing

Genetic information is stored in nucleotide pairs, i.e., adenine (A) with thymine (T), and guanine (G) with cytosine (C). From this, all genes are written as sequences of these four nucleotides. Sometimes, during cell mitosis, DNA is not duplicated correctly, and mutations may occur; in some cases, entire parts of a chromosome may be lost. Some mutations may be silent and produce no observable effects, while others may be highly oncogenic.

In neurological oncology, two mutations are of particular importance: the IDH [91] and PTEN [46] genes. Mutation in the IDH1 or IDH2 genes affects cellular metabolism, and as a result, affected cells start to produce 2-hydroxyglutarate, a metabolite that is oncogenic. This mutation is present in IDH-mutant gliomas (WHO grades 2, 3, and 4). IDH-mutant gliomas of grades 2 and 3 have a tendency to advance in time into higher-grade gliomas. The PTEN gene acts as a crucial tumor suppressor gene in a cell. When this gene is not properly active or is totally absent, e.g, as a result of a chromosome 10q deletion, the PI3K/AKT/mTOR signaling pathway becomes hyperactive [46], which in turn is highly oncogenic. This mutation promotes the worst-prognosing brain tumor, glioblastoma IDH-wildtype.

In [92], the authors use TCGA (The Cancer Genome Atlas) SCNA (somatic copy number alteration) data from 786 adult diffuse gliomas to predict the IDH mutational status. First, the authors converted the genetic data to a 39-dimensional chromosome arm-level data representation. Next, after filtering out the oligodendrogliomas, they trained and evaluated numerous machine learning classifiers that, based on genetic data, predicted the presence of IDH mutation in the remaining diffuse astrocytic gliomas. Finally, the logistic regression classifier was chosen. The resulting classifier provides near-perfect classification with an AUC of 0.99.

In genetic research, one often uses transcriptome sequencing applied to a single cell to gain insight into the origin of a tumor and measure the levels at which certain genes are expressed. In the most frequently observed approaches, cells are aggregated into sub-population clusters based on their gene expression, signaling, and disease-promoting changes. This clustering is commonly performed using K-means or hierarchical clustering approaches. This, in turn, can be challenging as one deals here with data that is sparse and highly dimensional. The authors of [93] describe a new, multi-stage approach that, by combining classical machine learning and deep learning, achieves very good results using ARI, NMI, and PUR metrics. The final module of the described approach utilizes a deep fuzzy clustering method, which, through a deep neural network, estimates cluster assignments.

Another multimodal clustering method for data from scRNA-seq and scATAC-seq has been described in [94]. Here, similarly to the previously mentioned paper, the authors focus on clustering analysis of transcriptome data. The described scEMC process takes as input scRNA and scATAC data, denoises them using a dedicated autoencoder, and feeds the result into a transformer-based neural network. The output of the transformer network is put through the skip aggregation network. The resulting embeddings enable authors to obtain high-quality cell representations that can achieve unsupervised clustering with high accuracy. The results, similar to those in [93], are evaluated using ARI, NMI, and PUR metrics.

#### 2.5.3. Summary

There are many advanced decision support systems used in histopathology and genetic analysis. Especially in histopathology, where the goal is the analysis of the image data of biological samples, the neural network provides very good results. Machine learning is still developing in the genetic analysis field, as it is still mainly based on DNA sequencing and the search for known mutation patterns. All the computer-aided tools presented in this section that support neurosurgical procedures are summarized in Table 3.

## 3. Decision Support in Vascular Neurosurgery

Brain arteriovenous malformations (AVMs) often remain clinically silent and undiagnosed for an extended period of time. They are frequently discovered incidentally during CT or MRI scans performed for unrelated reasons, or when they manifest with neurological symptoms. Precise identification and classification of such malformation are critical, as clinical strategies may vary significantly. Some lesions require only observation, while others, such as an aneurysm, can pose an immediate, life-threatening risk. At the same time, distinguishing between different types of vascular malformations can be challenging, even for experienced radiologists. In response to this clinical demand, a growing number of artificial intelligence models have been proposed to support the diagnostic process. Unfortunately, the application of AI for detecting and classifying vascular abnormalities in neurosurgery remains relatively underdeveloped compared to other sub-specialties. Therefore, these approaches are presented collectively within a single subsection.

### 3.1. Intracranial Aneurysm

Large screening programs can help to assess the risk of developing an intracranial aneurysm (IA). However, they require analyzing copious amounts of data, which is a time-consuming task. In [98], Heo et al. used machine learning to support and speed up the process. They used four approaches: logistic regression, Random Forest, scalable tree boosting system, and deep neural networks. Models were trained on a dataset comprising 21 features from over 400 thousand subjects screened in South Korea. The boosting system performed best with an ROC AUC of 0.765. However, other methods scored similarly with ROC AUC of 0.762 for logistic regression, 0.757 for a random forest, and 0.748 for a deep neural network comprising seven layers. With the advent of large language models, such a study could be addressed by analyzing whole reports with such advanced architectures, most likely leading to even better performance.

Several methods have been proposed for the detection and segmentation of IAs, which can be a tedious task when performed manually. Zhu et al. trained 3D UNet, VNet, and 3D Res-UNet on the dataset of 101 sets of cranial computed tomography angiography (CTA) comprising 140 IA cases [99]. Models achieved an average voxel-wise recall of 79.7% for 3D UNet, 77.1% for VNet, and 68% for 3D Res-UNet. Although medical practice would require a much higher recall, such models might still be helpful in supporting the decision made by a neurosurgeon.

Detection and segmentation of IA using CTA images were also tackled by Yang et al. [100]. Their integrated deep model using the nnU-net architecture was trained on the dataset comprising 1182 patients with unruptured IA and 578 control subjects. The reported model achieved an accuracy of 97% in detecting IA and a Dice score coefficient of 0.90 for IA segmentation, which constitutes a significant accomplishment.

Another method, SrtokeNet, was proposed by Irfan et al. [101]. It is an end-to-end approach for aneurysm segmentation and rupture risk prediction. Aneurysm segmentation is performed on digital subtraction angiography (DSA) images using a fully connected convolutional neural network. This step is followed by the extraction and fusion of various features using a multidisciplinary approach. This includes deep features, geometrical features, Fourier descriptors, and shear pressure on the aneurysm wall. The rupture risk is assessed as mild, moderate, severe, and critical by the Decision Tree classifier. Performance was measured by comparing the system output to expert neurosurgeon annotations, and the system performed well with 85% accuracy.

Abdullah et al. [102] proposed a DeepInfusion model for the segmentation of IA based on DSA images. To improve detection and segmentation, the authors incorporate expert knowledge into the model as an attention mechanism. Several experiments were performed to apply the infusion to different layers. The final model achieves accuracy above 90%, making it suitable for clinical applications.

Differentiating between ruptured and unruptured intracranial aneurysms is extremely important in neurosurgical practice. A ruptured aneurysm typically causes subarachnoid hemorrhage associated with high mortality, requiring immediate intervention. In contrast, unruptured aneurysms are often detected incidentally and may not necessitate urgent intervention. Their management involves a careful assessment of rupture risk versus the potential complications of preventive procedures. Misclassification can lead to either delayed life-saving treatment or unnecessary exposure to surgical risks. To support the decision process, Silva et al. [103] trained three ML models to discern between ruptured and unruptured aneurysms. The data used for training and evaluation comprised information derived from radiology reports and medical history for a total of 845 aneurysms in 615 patients. Models scored well, with ROC AUCs of 0.77 for linear SVM, 0.78 for radial basis SVM, and 0.81 for a Random Forest classifier, indicating that these classical methods are able to classify aneurysms and identify features associated with rupture correctly.

### 3.2. Other Vascular Conditions

As vascular neurosurgery is not limited to aneurysm diagnosis and treatment, AI-based support systems have also been developed for other diseases and pathological conditions. Such methods may improve the diagnosis of cerebral cavernous malformations (CCM), as indicated by Kim et al. [104]. This group of researchers developed a diagnostic pipeline using a commercially available AI algorithm (Medical Insight + Brain Hemorrhage, SK Inc. C&C, Seongnam, Republic of Korea) [105] to assist in pinpointing possible hemorrhage locations by generating heatmaps and probability scores through slice-wise and patient-wise analysis. Their findings indicate that AI-assisted diagnosis was more accurate (accuracy of 86.92%) than the traditional approach (79.86%) and that the difference is statistically significant with p<0.001. In [106] Jabal et al. present a quantitative and explainable model, allowing for the prediction of intracranial hemorrhage and functional outcome in CCM patients. They have developed several classifier models that interpret clinico-radiologic and radiomic features extracted from MRI scans. The dataset used consisted of scans from 366 adults, of whom 181 were subjects affected by CCM. The authors tested various classical machine learning models, including Linear Regression, Decision Tree, Random Forest Classifier, AdaBoost, Gradient Boosting, and a multilayer perceptron. For hemorrhage prediction, linear regression achieved the best accuracy with an ROC AUC of 0.86. The multilayer perceptron achieved the best results with an ROC AUC of 0.74 for predicting a Rankin Scale score of ≥2, which is used to assess the functional outcome of patients. The authors tested many classical models and a multilayer perceptron, but have not experimented with advanced architectures that could be derived directly from the MRI images, or with multimodal approaches combining derived features with raw images.

In [107], Akiyama et al. adopted a transfer learning approach using the VGG16 architecture [34] to distinguish between patients with moyamoya disease [108], atherosclerotic disease, and healthy subjects. This model identifies patients with moyamoya disease based on T2-weighted MRI slices from the basal cistern, basal ganglia, and centrum semiovale, achieving accuracies of 92.8%, 84.8%, and 87.8%, respectively.

All the computer-aided tools presented in this section that vascular neurosurgery procedures are summarized in Table 4.

## 4. Decision Support in Functional Neurosurgery

The aim of functional neurosurgery is the modification of the physiological activity of selected parts of the brain. This can be achieved by means of implanting a permanent stimulating electrode into the brain or by making purposeful lesions in such areas by applying heat or cold. In most cases, the targets of functional neurosurgeries are small, located deeply within the brain, and in areas close to parts of the brain performing critical functions. Misdetection of the target of the surgery by a few millimeters may have very serious adverse effects. Moreover, if the surgery is to make a purposeful lesion, adverse results will be irreversible. All the above make such surgeries difficult and without a margin for error. In such a situation, a decision support system that helps to locate the target of the surgery precisely may be very helpful.

### Decision Support for Deep Brain Stimulation for Parkinson’s Disease

In the case of Deep Brain Stimulation (DBS) for Parkinson’s Disease, the target of the surgery is a small structure called the Subthalamic Nucleus (STN). It is a few millimeters in diameter and is located deep within the brain, ventral to the thalamus. While the goal is the stimulation of the anterior part of this structure, its posterior part or other adjacent brain structures must not be stimulated [109]. It is therefore important to precisely pinpoint the location of the STN within the brain. One method that can be used for the precise localization of the STN is the microrecording procedure. This procedure is part of the DBS surgery and locates the STN by means of its distinct physiological activity. In this approach, preoperative CT and MRI scans are used to approximate the localization of the STN. Later, during the DBS surgery, several parallel thin recording microelectrodes are inserted into the patient’s brain towards the expected localization of the STN. When these electrodes are positioned approximately ten millimeters above the dorsal border of the STN, the recording procedure begins. Electrodes record the physiological electrical activity of the brain tissue for about ten seconds, and then are advanced by one millimeter. This recording and advancing is repeated until the electrodes reach a depth below the ventral border of the STN. As the STN has a distinct physiological activity, the neurosurgeon can discriminate which of the recordings have been registered within the STN. From this, one can tell which of the recording electrodes at which depths passed through the STN. Analysis of such recordings is, however, complex and subjective, so various decision support systems have been developed to help with this task.

In [110], the author describes the mathematical and machine learning foundations of a decision support system that was designed for discrimination between STN and other recordings. The described solution does not facilitate deep learning; it only involves hand-crafted attributes and classical machine learning. Still, it achieves very good results and provides explainability and interpretability, which are much appreciated in clinical medicine. In the paper, an original wavelet-based method for the removal of the artifacts that are ever-present in DBS recordings is also described. The obtained results are excellent, with the sensitivity and specificity being both above 96%, and the ROC AUC being above 0.99. These results are among the best described in the literature.

**Figure 2:**
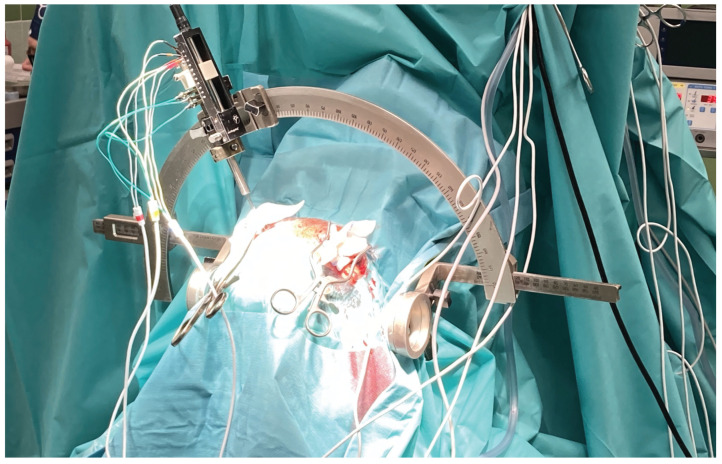
The microrecording procedure during DBS surgery.

In [11], the authors provide a deep learning-based solution for detection of recordings acquired within the STN. The solution described in [110] requires removal of the artifacts prior to the calculation of the attributes and following classification. Artifacts must be removed as their presence tends to produce false-positive classification of the recordings from outside of the STN as STN ones. The neural network described in this paper does not require such preprocessing. Using a modified ResNet architecture with additional attention layers that act in the temporal domain, the network itself detects and eliminates artifacts. For the test data, the network achieved sensitivity 89% and specificity 85% with AUC 0.94. The models were trained and tested on the Mendeley Dataset (https://data.mendeley.com/datasets/tr93krswn2/1, accessed on 13 November 2023). The dataset contains 4650 DBS recordings acquired during 46 DBS surgeries for Parkinson’s Disease (Figure 2).

## 5. Future Directions for DSSs in Neurosurgery

Future development of DSSs in neurosurgery should prioritize several key directions. First, there is a growing emphasis on creating anatomically grounded and interpretable models that integrate seamlessly into clinical workflows. These systems must not only classify or predict outcomes but also provide actionable insights that align with surgical reasoning and patient-specific contexts. With the development of novel architectures for textual or imaging data types, one might try to apply more complex models to achieve better performance.

A highly promising direction in the development of AI-based decision support systems in neurosurgery involves the integration of multimodal data sources. These may include structural and functional imaging modalities such as MRI and CT, engineered features derived from raw imaging data, patient medical history, laboratory results, and genomic profiles. This integrative approach enables the construction of more holistic and personalized models capable of capturing the complex interplay of anatomical, physiological, and clinical variables.

**Figure 3:**
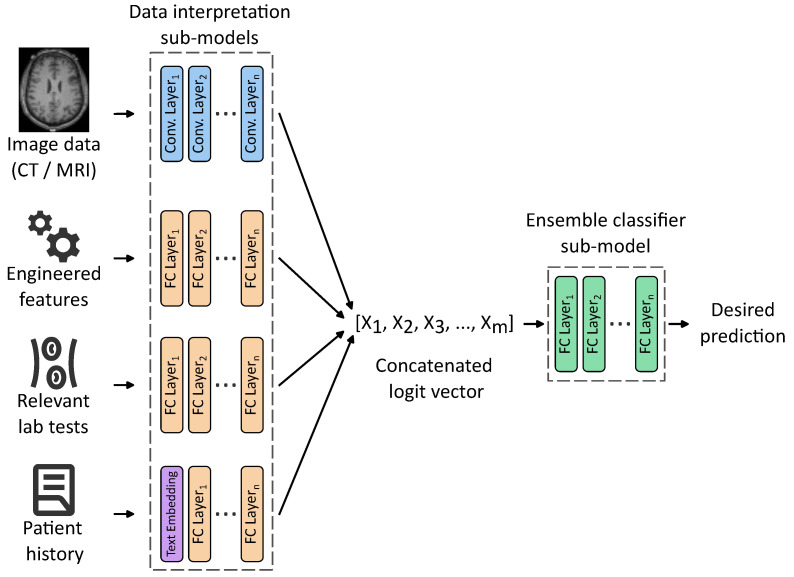
An idea of a multimodal ensemble network allowing us to train weights of input processing sub-models, together with an ensemble classifier. For simplicity, only the main layers are shown with activation functions, dropouts, max pooling, etc., hidden.

Figure 3 exemplifies an idea of such architecture, comprising data-processing sub-models trained together with an ensemble classifier sub-model. Each data processing sub-model is designed to process a distinct modality using a tailored set of weights organized into one or many layers of various kinds, that are disjoint with other sub-models. For instance, 2D and 3D convolutional layers are well-suited for interpreting raw imaging data, while fully connected layers can effectively handle structured numerical features. Patient history, typically in an unstructured text form, can be included using appropriate text embeddings, calculated with either classical methods such as bag-of-words or Word2Vec, or more advanced word embeddings generated by large language models (LLMs).

Each of these sub-models outputs a logit vector representing high-level features extracted from its respective input. These vectors are then concatenated into a unified representation, a meta-feature vector serving as an input to the ensemble classifier sub-model responsible for computing the final prediction. Crucially, the entire architecture can be trained end-to-end, eliminating the need to identify intermediate tasks and labels required to train each sub-model separately. This unified training paradigm enhances model coherence and allows for the discovery of latent cross-modal interactions that may be clinically relevant but difficult to capture through traditional modeling approaches.

Like in the case of almost any non-trivial machine learning solution, the creation of such a classifier is not a straightforward task. The illustration provides an idea for such a classifier, and in its implementation, many obstacles and challenges would have to be overcome.

When dealing with image data, one can obtain results from different devices that have varying spatial resolutions, color depths, and other characteristics. For example, a common practice in preparing image data prior to DBS surgery is the fusion of MRI and CT images. The MRI images provide better spatial resolution but are laterally distorted, while CT images, with lower resolution, retain proper image proportions.

Engineering the features is a time-consuming and challenging process. It requires close cooperation between engineers and medical practitioners to define proper, population-wide, useful discriminating attributes. Often, terms that are easy to express in spoken language and are easy to observe are extremely hard to express in formal mathematical definitions required by engineered features.

Lab tests must be normalized, as different laboratories often use different scales for test results. Depending on the laboratory, certain tests might be in different units, scales, or even completely absent. Analysis of a patient’s history is a chapter in itself. This is often non-structured data, and the possible application of LLM introduces additional risks.

Training such a vast network could also be challenging due to its size and issues with gradient backpropagation. It would have to be trained partially with the possible addition of residual connections.

**Figure 4:**
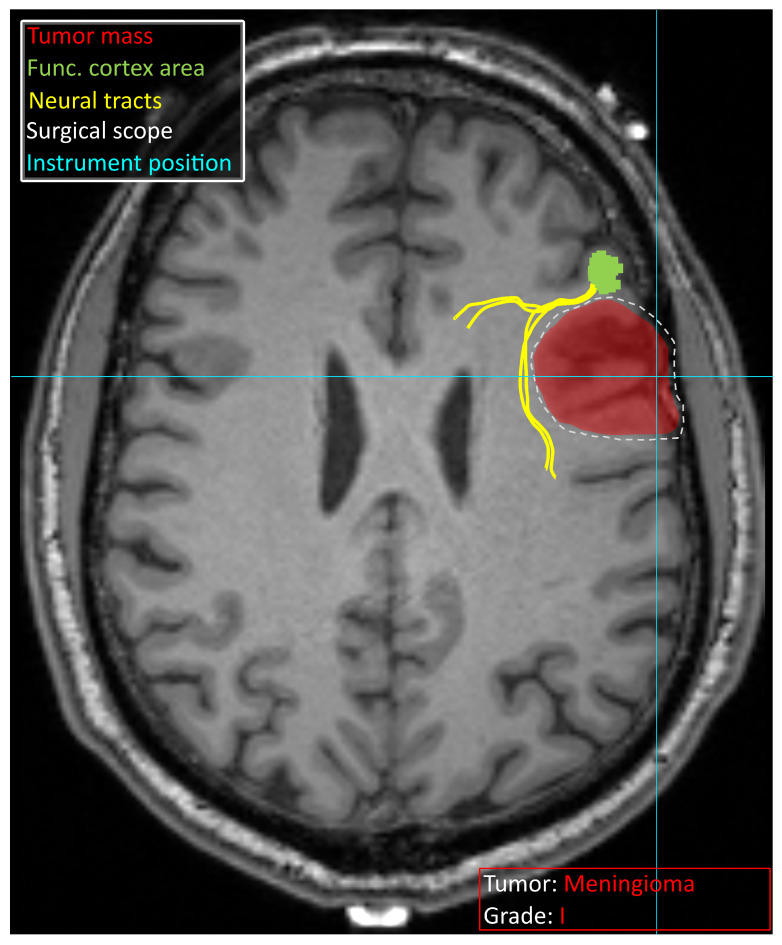
Schematic overview of information that a potential decision support system can provide to a neurosurgeon. The identified tumor is marked in red, and the tumor type and grade are specified. The functional area of the cortex identified with fMRI is visible in green, with neural tracts leading to it in yellow. The surgical intervention site suggested based on the available data is marked with a white dashed line. Bright blue lines crossing indicate an instrument position derived from neuronavigation. Schematic vision was provided on a T1 MRI scan obtained from [111].

Decision support systems in neurosurgery are currently characterized by a wide array of specialized models, each tailored to a specific task and data modality. While this diversity provides neurosurgeons with a rich set of tools to support their daily clinical practice, it also introduces a significant cognitive and operational burden as they have to navigate multiple tools requiring distinct input formats and often complex preprocessing pipelines. Therefore, the development of DSSs in neurosurgery should not focus solely on improving the predictive accuracy of individual models. Equally important is the development of integrated platforms capable of aggregating, orchestrating, and synthesizing outputs from multiple models, while presenting the results in a clinically meaningful and user-friendly interface. Such systems should deliver comprehensive and context-aware information to the neurosurgeon both during preoperative planning and during the surgery. Figure 4 schematically illustrates a vision of such a system, designed to present multimodal information directly on the screen, minimizing the need for manual data handling or document review. This includes real-time visualization of imaging data, anatomical annotations, risk stratification scores, and patient-specific recommendations derived from predictive models. By consolidating these insights into a single, intuitive interface coupled with neuronavigation, the system aims to provide as much information as needed in real time for safer and more efficient surgeries.

## 6. Summary and Discussion

This comprehensive review has explored the diverse applications of machine learning, particularly artificial intelligence and deep learning, in supporting clinical decision-making across various subspecialties of neurosurgery.

As both neurosurgery and AI are relatively new specialties in medicine and computer science, respectively, their combination creates new ground for scientific advances that are beneficial to both specialties. Both neurosurgery and computer science are extremely broad specialties, and as such, there is always a risk that while conducting a review, one might approach the subject in too broad or too narrow a focus. A too-broad approach might result in the review being insufficiently extensive in some areas, while a focus on specific areas might overlook others entirely. In this work, the authors aimed for a balanced approach to present various areas of applicability of the DSS in neurosurgery, without exclusively focusing on a single branch, such as vascular or functional neurosurgery.

Additionally, in some areas of scientific advancement, the same solution to a certain problem may be of equal importance in computer science as it is in medicine. Certain approaches for glioma classification offer a significant advancement in computer vision, while from the clinical practitioner’s point of view, they do not provide much advancement to current medical knowledge. On the other hand, certain DSSs that do not advance significantly in computer science but propose novel uses of existing algorithms might prove to be extremely useful in clinical medicine.

There are also still discussions in both sciences as to the goals this scientific cooperation should aim towards. Some studies look for a DSS that would just be a tool that leaves interpretation of the results to medical professional. Others look for dialogue system like one provided by large language models, and in [112] they even declare that ’the ultimate goal of AI is to create consciousness’. This is the point of view one should certainly be careful about. While intelligent tools are beneficial, the results of application of a tool with its own conscious agenda might be risky, especially in such critical applications as neurosurgery.

To emphasize how new a concept AI is, one might chose the relation between machine learning, deep learning and AI. In [112] the AI correctly includes machine learning which in turn includes deep learning. Meanwhile some studies tend to identify AI as a deep learning-related term and treat it as a subsection of broadly understood machine learning.

The described methods are summarized in Table 5. While numerous studies have investigated the potential of deep neural networks, their performance often does not significantly surpass that of classical ML methods such as logistic regression or support vector machines. Neural networks tend to excel in modeling complex, nonlinear relationships, especially in high-dimensional imaging data, but their superiority is often context-dependent and not universally observed across all neurosurgical tasks.

Despite promising results, current AI models still require substantial refinement to meet the stringent accuracy demands of medical applications. In neurosurgery, where diagnostic and therapeutic decisions can have profound consequences, even marginal improvements in predictive accuracy on the order of 3–4 percentage points can translate into meaningful clinical benefits. This underscores the importance of continued efforts to enhance model precision, robustness, and generalizability. Future research should focus on leveraging more advanced architectures trained on increasingly large and diverse datasets, including those available in public repositories. Importantly, these experiments must not compromise model interpretability, as explainability remains a critical factor in clinical adoption and can offer valuable insights to neurosurgeons.

The review also discusses the potential of multimodal approaches, particularly architectures composed of sub-models. These systems would allow the processing of heterogeneous input data of various types (imaging, numerical, textual, etc.) using modality-specific layers and sub-models. The outputs of these sub-models are then integrated via an ensemble classifier, enabling a unified decision-making process. Such architectures offer a promising pathway toward more comprehensive and personalized decision support.

Beyond improving individual models, we emphasize the importance of designing integrated platforms that consolidate outputs from various analytical tools into a single, coherent system. These platforms should support both preoperative planning and intraoperative guidance, ideally through integration with neuronavigation systems.

In conclusion, while AI has already begun to transform neurosurgical practice, its full potential lies in the development of integrated, interpretable, and clinically embedded decision support systems.

Besides interpretability, the further development must also focus on explainability, especially in deep learning applications. Explainability not only provides assurance regarding the given decision but also enables medical staff to learn from the provided solutions.

There are ongoing legal debates regarding the responsibility for medical errors caused by software solutions. There are already cases in history when a software error was responsible for the death of a patient [113]. One cannot blame a medical practitioner for not implicitly trusting a black box solution that does not provide any interpretability or explainability. In the case of medical error, he/she might be the only one to blame.

These systems must be designed not only to improve predictive performance but also to align with the practical needs of neurosurgeons, ultimately contributing to better patient outcomes.

## Figures and Tables

**Table 1:** Summary of selected computer-based tools supporting tumor detection and classification.

Authors	Scope	Method	Input	Metrics	Dataset
Jia et al. [25]	Brain tumor detection	SVM	MRI slices	Acc = 98.51%	Multi-parametric MRI images
Anantharajan et al. [27]	Brain tumor detection	SVM	MRI slices	Acc = 97.93% sensitivity = 92% specificity = 98%	Kaggle open data 255 T1-mode MRI
Mittal et al. [28]	Brain tumor segmentation	Hybrid technique: SWT + RF + GCNN	MRI slices	MSE = 0.001 sensitivity = 98.23% precision = 98.81%	BRAINIX medical images
Abiwinanda et al. [29]	Brain tumor classification (3 classes): glioma, meningioma, pituitary	CNN	MRI slices (512 × 512)	Acc = 84.19%	Figshare [32] (3064 CE-MRI)
Badža et al. [30]	Brain tumor classification (3 classes): glioma, meningioma, pituitary	CNN	MRI slices (256 × 256)	Confusion matrices Acc = 88.48%	Figshare [32] augmented dataset (9192 CE-MRI)
Rehman et al. [31]	Brain tumor classification (3 classes): glioma, meningioma, pituitary	CNN: AlexNet, GoogLeNet, VGG16	MRI slices (512 × 512)	Acc = 98.69%	ImageNet [33], Figshare [32]
Li et al. [35]	Glioma grading classification	3D-ResNet101	MRI image	Acc = 83% F1 score = 83% AUC = 0.89	TCIA [38], 708 glioma patients from 2nd Hospital of Lanzhou University

**Table 2:** Summary of selected computer-based tools supporting preoperative planning.

Authors	Scope	Method	Input	Metrics	Dataset	Characteristics
Kuan et al. [51]	fMRI-based identification of brain regions subserving language	RF, WEASEL, TSFresh, RISE, sTSF, ARSENAL, Inception	fMRI EPI sequences, T1 MRI 256 × 256	AUC = 0.97, ± = 0.03	Patients of Royal Brisbane and Women’s Hospital	Evaluation of different ML/DL methods in predicting language related brain regions
Luckett et al. [59]	Mapping of resting-state networks in the brain	3D CNN	T1w MRI 256 × 256; RS-fMRI scans, voxel 3 4 mm^3^; T1w MRI voxel 1 mm^3^; T2w MRI voxel 1 mm^3^	LAN = 96.9%, MOT true-positive rate = 96.3%	2252 patients from [73,74]	Demonstrates the utility of DL for accurate mapping of eloquent cortex using a reduced amount of RS-fMRI data
Neher et al. [61]	Tracking fiber pathways	RF + boosting by voting	3D gradient data	Valid connections = 93%, bundle coverage rate = 94%	nitrc.org/projects/diffusion-data; https://tractometer.org/ismrm2015/home/; www.humanconnectome.org/data	RF-based fiber tractography using neighborhood information.
Heker et al. [62]	Segmentation of anatomical fiber tracts	AdaBoost, Viola Jones algorithm	DWI MRI 3D data	FP = 0.44%, detection rate = 98.77%	Human Connectome Project	Novel approach to the automatic segmentation of brain tracts
Zhang et al. [63]	Analysis of whole brain white matter for autism spectrum disorder detection	SVM with polynomial kernel	DWI MRI 3D data	Acc = 78.33%	149 pediatric male children (70 wist ASD) from Center for Autism Research, Children’s Hospital of Philadelphia	Novel method of autism spectrum detection from DWI MRI data
Jörgens et al. [64]	Step-by-step prediction of streamline tractography from DWI MRI data	NN composed of fully connected layers	DWI MRI 3D data	Angular error below 5.5°	ISMRM 2015 tractography challenge data [75]	Streamline prediction using NN with linear interpolation of DWI data
Wegmayr et al. [65]	Entropy-based approach to fiber tractography	Maximum Entropy Principle, conditional distribution, NN composed of fully connected layers	DWI MRI 3D data	Valid bundles = 23 out of 25; valid connections = 51%	Human Connectome Project	Probabilistic framework for ML approaches to fiber tractography
Wegmayr et al. [66]	Entropy-based approach to fiber tractography	Maximum Entropy Principle, conditional distribution, entropy regularization, annealing, NN composed of fully connected layers	DWI MRI 3D data	Valid bundles = 24 out of 25; valid connections = 52%	Human Connectome Project	Probabilistic framework for ML approaches to fiber tractography
Wasserthal et al. [67]	White matter bundle segmentation	CNN (U-Net architecture)	DWI MRI 3D data	Dice score (CST, SLF) close to 0.85	ISMRM 2015 tractography challenge data [75]	Human-comparable performance achieved on all bundles
Gupta et al. [68]	Detection and clustering of white matter fibers	2D CNN	DWI MRI 3D data	Not given	Not specified	Sampling of fiber data with replacements for application of CNN
Gupta et al. [69]	Automatic clustering of white matter fibers	2D CNN	DWI MRI 3D data	Acc above 97%	Parkinson’s Progression Markers Initiative (226 individuals)	False-positive fibers are removed from a fiber bundle segmented using ROI-based segmentation tools
Wasserthal et al. [70]	White matter fiber segmentation	CNN CNN (U-Net architecture)	DWI MRI 3D data	Outperforms the reference methods by 14 Dice points	Proprietary dataset [76]	Very good results for low-quality datasets
Kumar et al. [71]	White matter fiber segmentation	Fully connected CNN with skip connections and spatial attention, Gray Wolf Optimization to tune CNN classifier	DWI MRI 3D data	Acc = 97.10%, sensitivity = 95.74%, F1 = 94.79%	Human Connectome Project	Novel, efficient classifier
Korycinski et al. [72]	Fiber tracking	Hybrid technique: fully connected CNN + A* path search algorithm	DWI MRI 3D data	Mean Euclidean Distance (MED) to EuDX below 10	Human Connectome Project	Combination of deep learning with classical ML

**Table 3:** Summary of computer-aided tools supporting neurosurgical procedures.

Authors	Scope	Method	Input	Metrics	Dataset
**Intraoperative neuronavigation**					
Mazzucchi et al. [77]	Multimodal imaging for improving intraoperative neuronavigation	Fusion of T1w, T2w, DTI, Flair and ultrasound	MRI: T1w, T2w, DTI and Flair modalities; intraoperative ultrasound	Descriptive	Proprietary
Guo et al. [78]	Intraoperative MRI and ultrasound in diffuse glioma surgery	Fusion of T1w, T2w, DTI, DWI and ultrasound	MRI: T1w, T2w, DTI and DWI modalities; intraoperative ultrasound	Increased extent of resection, more cases with a total resection; increased operative time	Proprietary
Wei et al. [79]	Intraoperative MRI navigation for glioma resection	Segmentation dictionary learning algorithm	MRI: T1, T2, Flair, DTI and BOLD modalities	Descriptive	Proprietary
**Stereotactic biopsy guidance**					
Şahin et al. [80]	Trajectory selection in stereotactic brain biopsy	3D Residual U-Net	CT, MRI, MRA	Descriptive	Proprietary
**Intraoperative brain mapping**					
Ishankulov et al. [81]	Prediction of post-operative speech impairment based upon evoked potentials	RF, logistic regression, SVM	Cortico-Cortical Evoked Potentials	Descriptive	Proprietary
**Post-operative/traumatic intensive care**					
Schweingruber et al. [82]	ICP hypertension prediction in neuro ICU patients	LSTM	ICP monitoring	Descriptive	Proprietary
Petrov et al. [84]	Prediction of an ICP crisis in patients with TBI	RF, XGBoost, LGBM	ICP monitoring	Acc = 88%, AUC 0.87	Proprietary
**Histopathology**					
Ker et al. [86]	Automatic brain histological classification	CNN (Inception V3 model)	Histology images 1600 × 1200	Normal brain vs. high-grade glioma: F1 = 100%; normal brain vs. glioma: F1 = 99%;	Proprietary from Department of Pathology at Tan Tock Seng Hospital
Orringer et al. [88]	Fast intraoperative histology of unprocessed surgical specimens	Fully connected network (MLP)	SRS microscope images	Lesional vs. non-lesional: specificity = 94.1%, sensitivity = 94.5%	Proprietary
Quan et al. [89]	Few-shot pathology image classification	Transformers, ResNet	Histology images: 150 × 150, 224 × 224, 768 × 768	Acc = 85.87%, AUC = 0.98	CRCTP [95], NCTCRC [96], LC25000 [97]
Nan et al. [90]	DL patterns for whole-slide image diagnosis	Vision Transformer, ResNet50	Histology images 224 × 224	Acc = 93%, AUC = 0.984	Proprietary from First Affiliated Hospital of China Medical University
**Genetic testing**					
Nuechterlein et al. [92]	Prediction of IDH mutational status in adult diffuse glioma	PCA, various scikit-learn classifiers	Genome-wide somatic copy number alteration, The Cancer Genome Atlas	Descriptive	https://xena.ucsc.edu, https://gdc.cancer.gov
Hu et al. [93]	Deep single-Cell multiview fuzzy clustering	KNN, graph random walk, node2vec, transformers	Transcriptome data	Descriptive	https://github.com/satijalab/seurat-data, https://www.10xgenomics.com/resources/datasets
Hu et al. [94]	Multi-modalclustering of scRNA-seq and scATAC-seq data	Transformers, embeddings, KL divergence, deep k-means clustering	scRNA-seq and scATAC-seq data	ARI, NMI metrics	https://www.ncbi.nlm.nih.gov/geo, https://www.10xgenomics.com/resources/datasets, https://github.com/YosefLab/totalVI_reproducibility

**Table 4:** Summary of selected computer-based tools supporting vascular neurosurgery.

Authors	Scope	Method	Input	Metrics	Dataset
**Intracranial aneurysm treatment**					
Heo et al. [98]	Prediction of intracranial aneurysm risk	Logistic regression, RF, scalable tree boosting system, and ANN	Tabular data from NHIS-NSC	AUC = 0.76	NHIS-NSC Korean database
Zhu et al. [99]	Detection and segmentation of intracranial aneurysms with a small sample size	3D UNet, VNet, 3D Res-UNet	CTA images of 101 patients with 140 aneurysms	Sensitivity = 80.9%	Proprietary from First Affiliated Hospital of Xi’an Jiaotong University
Yang et al. [100]	Detection, segmentation and analysis of intracranial aneurysms using CT and angiography	CNN (U-Net architecture)	CT, angiography	Acc = 97%	Proprietary
Irfan et al. [101]	Segmentation and rupture risk prediction of intracranial aneurysm	CNN (U-Net architecture), decision tree	DSA images: 128 × 128, 256 × 256, 512 × 512	Acc = 68& 87%, precision: 65% 84%, sensitivity = 67% 81%	2D Digital Subtraction Angiography (DSA) images
Abdullah et al. [102]	Segmentation of intracranial aneurysms	Infusion-based AI model (U-Net)	DSA images: 128 × 128, 256 × 256, 512 × 512	Acc = 99%, F1 = 63.6%	2D Digital Subtraction Angiography (DSA) images
**Vascular conditions treatment**					
Kim at al. [104]	Differentiation of cavernous malformation and acute intraparenchymal hemorrhage	CNN	CT images	AI-assisted results (accuracy, sensitivity, specificity) significantly better than unassisted (*p*-value < 0.001)	Proprietary, not public
Yun et al. [105]	Automatic detection algorithm for acute intracranial haemorrhage	CNN, RNN, VAE, GAN	CT images	AI-assisted results (accuracy, sensitivity, specificity) significantly better than unassisted (*p*-value < 0.001)	Proprietary
Jabal at al. [106]	Prediction of outcomes in cerebral cavernous malformations	SHapley Additive exPlanations (SHAP)	MRI: T2w, FLAIR modalities	*p*-value results confirm quality of predictions	Proprietary
Akiyama et al. [107]	The Moyamoya Disease diagnosing.	Xception, VGG 16, VGG19, Inception V3, ResNet, DenseNet	MRI, MRA	Acc = 99%	Proprietary from Sapporo Medical University Hospital

**Table 5:** Summary of decision support methods in neurosurgery, categorized by application domain and methodological approach.

Application Type	Method Type	References
Diagnosis	Machine Learning	[25,27,28,44,45,92,98,106]
	Deep Learning	[23,27,28,29,30,31,35,42,43,89,90,93,94,98,99,100,101,102,103,104,105,106,107]
Preoperative	Machine Learning	[51,61,62,63]
	Deep Learning	[23,51,59,64,65,66,67,68,69,70,71,72]
Intraoperative	Machine Learning	[79,81,110]
	Deep Learning	[11,77,78,80,88]
Postoperative	Machine Learning	[84]
	Deep Learning	[82,86]

## Data Availability

The original contributions presented in this study are included in the article. Further inquiries can be directed to the corresponding author.

## Abbreviations

The following abbreviations are used in this manuscript:

Acc	Accuracy
AI	Artificial Intelligence
AVM	Arteriovenous Malformation
CCEp	Cortico-cortical Evoked Potential
CCM	Cerebral Cavernous Malformations
CNN	Convolutional Neural Network
CT	Computed Tomography
CTA	Computed Tomography Angiography
DBS	Deep Brain Stimulation
DSA	Digital Substraction Angiography
DSS	Decision Support System
fMRI	Functional Magnetic Resonance Imaging
IA	Intracranial Aneurysm
ICP	Intracranial Pressure
LLM	Large Language Model
ML	Machine Learning
MRI	Magnetic Resonance Imaging
ROC AUC	Receiver Operating Characteristic Area Under the Curve
rs-MRI	resting-state MRI
SCS	Spinal Cord Stimulation
STN	Subthalamic Nucleus
SVM	Support Vector Machine
TBI	Traumatic Brain Injury
TCGA	The Cancer Genome Atlas
